# A Global Fundamental Matrix Estimation Method of Planar Motion Based on Inlier Updating

**DOI:** 10.3390/s22124624

**Published:** 2022-06-19

**Authors:** Liang Wei, Ju Huo

**Affiliations:** School of Electrical Engineering and Automation, Harbin Institute of Technology, Harbin 150001, China; weiliangts@163.com

**Keywords:** planar motion, inlier updating, triple-view constraint, global fundamental matrix, 3D reconstruction, visual localization

## Abstract

A fundamental matrix estimation based on matching points is a critical problem in epipolar geometry. In this paper, a global fundamental matrix estimation method based on inlier updating is proposed. Firstly, the coplanar constraint was incorporated into the solution of the fundamental matrix to reduce the number of parameters to be solved. Subsequently, an inlier updating matrix was introduced according to the threshold of the epipolar geometry distance to eliminate the potential outliers and obtain a reliable initial value of the fundamental matrix. On this basis, we employed a four-point iterative method to estimate the fundamental matrix and make it satisfy the rank constraint at the same time. Finally, the epipolar geometry in binocular vision was extended to triple-view, and the fundamental matrix obtained in the previous step was globally optimized by minimizing the coordinate deviation between the intersection point and feature point in each group of images. The experiments show that the proposed fundamental matrix estimation method is robust to noise and outliers. In the attitude measurement, the maximum static error was 0.104° and dynamic measurement error was superior to 0.273°, which improved the reconstruction accuracy of feature points. Indoor images were further used to test the method, and the mean rotation angle error was 0.362°. The results demonstrate that the estimation method proposed in this paper has a good practical application prospect in multi-view 3D reconstruction and visual localization.

## 1. Introduction

The estimation of a fundamental matrix is a basic step for computer vision applications and is widely used in visual localization, camera calibration [1,2], object recognition, motion analysis [3,4], 3D reconstruction [5,6], etc. Specifically, with the rapid development of 3D reconstruction technology, the demand and applications of 3D models in virtual reality, digital twin, the metaverse and other fields are rapidly growing [7,8]. The 3D reconstruction method using the images has been widely researched. In multi-view 3D reconstructions based on monocular vision, local invariance is used to detect image feature information and match feature points, which can be used to recover camera position, direction and scene structure. The matching of feature points and the relative pose between cameras directly affect the accuracy of reconstruction [9,10,11]. In robotics, the estimation of the fundamental matrix is the premise and foundation of visual localization. This accurately estimated fundamental matrix can be used to effectively construct the matching between feature points, and the relative pose between cameras is included in the fundamental matrix. Therefore, it is of great significance to develop a high precision and robust estimation method of the fundamental matrix [12,13].

The estimation accuracy of the fundamental matrix is mainly related to the extraction and matching accuracy of the feature points [14,15,16]. Among them, the extraction error is usually caused by the noise. When the feature points are not accurately matching, a small number of outliers will seriously affect the estimation accuracy of the fundamental matrix. To solve the above problems, many fundamental matrix estimation methods have emerged in recent years, including the linear method, iterative method, and robust method.

The linear method mainly includes a seven-point method, eight-point method, improved eight-point method, etc. [17,18]. These methods use least square and singular value decomposition to estimate the fundamental matrix by solving a set of linear equations. Under the conditions that feature point extraction and matching are accurate, linear methods are usually more efficient. However, the accuracy of linear methods is seriously affected when correspondences are abnormal. The iterative method can be divided into two categories: one is based on minimizing the epipolar geometric distance and the other is based on gradient [19,20]. Compared with the linear method, the iterative method improves the estimation accuracy and effectively reduces the influence of noise, although this method has a high computational complexity and is not suitable for cases with many outliers. Comparatively, the robust method has been widely used and studied for its advantages of eliminating outliers and strong anti-noise ability. Typically, M-estimator [21,22], LMedS [23,24] and RANSAC [25,26] are considered to be the most effective robust methods. To obtain reliable results, these methods usually screen matching points before calculating the fundamental matrix and take out the matching points with small geometric errors as inliers. Then, these inliers are used to estimate the fundamental matrix. Compared with these three methods, the M-estimator method reduces the influence of outliers by assigning different weights to each point, but this method has higher requirements for the initial values. The LMedS method uses the median distance from the point to the corresponding epipolar line to optimize the fundamental matrix, but this kind of method is quite time-consuming. The RANSAC method is used to estimate the fundamental matrix by iteratively selecting inliers. However, as the proportion of outliers increases, the computational efficiency significantly decreases.

To further improve the robustness and computational efficiency of the fundamental matrix estimation, many scholars made some improvements to the methods mentioned above. For example, Chum [27] proposed the randomized RANSAC method, which effectively reduces the amount of computation. The authors of [28,29] used different geometric constraints to iteratively solve the fundamental matrix. Moreover, Xiao et al. [30] proposed a fundamental matrix estimation method based on inlier set sample optimization. By adopting guided sampling and local optimization, this method can effectively deal with outliers. The above methods make up for the deficiency of the traditional robust method to some extent, but they also have a fatal problem, that is, the accuracy of these methods deteriorates sharply with the increase in outlier ratio. Later, Yan et al. [31] proposed a robust fundamental matrix estimation method based on epipolar geometric error criterion, which eliminated the outliers in the calculation process of the fundamental matrix and improved the calculation efficiency; however, the calculation accuracy of such methods needs to be further improved.

## 2. Related Work in Robotic Applications

Visual localization and multi-view 3D reconstruction of planar motion robots are important to indoor service robotic applications. Visual localization utilizes feature correspondences in the environment to recover the camera pose, and the relative pose between cameras can be used in 3D reconstruction. Among them, the fundamental matrix obtained by point correspondence can provide more constraints for camera pose estimation [32,33]. He et al. [34] systematically introduced the latest achievements of robot navigation in the literature and studied the simplified fundamental matrix of planar motion. Jiao et al. [35] introduced the fundamental matrix into 3D–2D correspondences to calculate the camera pose, which incorporated a planar motion constraint and enhanced the robustness of pose estimation. Choi et al. [36] proposed a 2-point non-iterative method based on epipolar geometry under a planar motion to estimate the camera pose. Choi et al. [37] proposed a minimal solver for pose estimation by establishing the fundamental matrix constraint and perspective projection function under a planar motion. Wang et al. [38] presented the importance of the fundamental matrix in robot active vision technology, and Dong et al. [39] devised a collaborative dense scene reconstruction method for multi-planar motion robot. These cases reduce the complexity of the algorithm and improve visual localization accuracy and 3D reconstruction accuracy by exploiting the fundamental matrix constraint under planar motion, but they do not conduct a thorough study on the solution of the fundamental matrix. The fundamental matrix is estimated by the traditional method.

Motivated by the above analysis, this paper proposes a minimal solution for estimating the fundamental matrix from point correspondence by taking the planar motion constraint into consideration. Among the existing methods, the linear method and iterative method are usually poor in robustness and not suitable for cases with many outliers. Although the robust method can eliminate outliers, it has the problem of unstable calculation when the outlier proportion is high. In addition, the number of iterations exponentially increases with the number of inliers required. When the camera moves on the plane, the accuracy of the traditional fundamental matrix estimation method decreases, and the required number of inliers is relatively large. The main contribution of this study is that it addresses the problem of a fundamental matrix estimation from multi-view images under planar motion, a robust null space estimation method is proposed. On this basis, the four-point iterative method is derived. Thus, the problem of rank constraint and local optimum caused by inlier random selection and an artificial threshold in the process of fundamental matrix estimation is successfully solved. Furthermore, the global optimization function of the fundamental matrix further improves the estimation accuracy.

The remainder of this paper is organized as follows. Section 3 introduces the epi-polar geometry in binocular vision and the traditional robust fundamental matrix estimation method. In Section 4, the global fundamental matrix estimation method based on the inlier updating of planar motion is proposed. Simulations and practical experiments are provided to verify the robustness and effectiveness of the proposed method in Section 5, and finally, the paper is concluded in Section 6.

## 3. Fundamental Matrix Estimation Method Based on RANSAC Method

**Figure 1:**
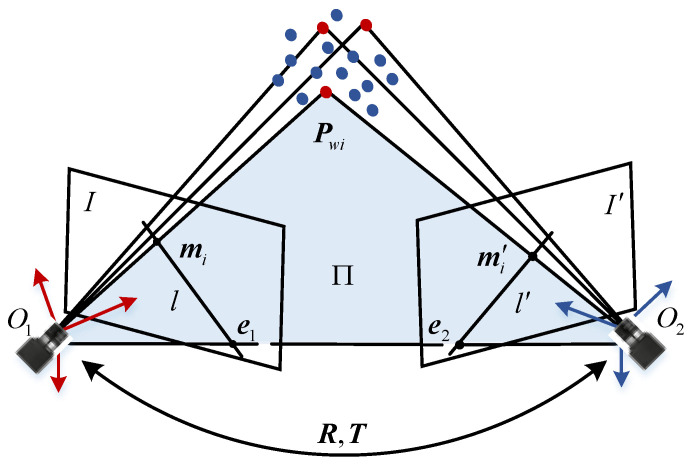
Epipolar geometry in binocular vision.

As shown in Figure 1, the projection points of a 3D point $P_{wi}$ on image planes are $m_{i}$ and $m_{i}^{\prime }$, respectively. $O_{1}$, $O_{2}$ indicate optical centers of the cameras, and the line between them is the baseline. The baseline intersects with image planes at two points $e_{1}$ and $e_{2}$, which are called epipoles. The plane formed by $P_{wi}$, $O_{1}$ and $O_{2}$ is defined as the epipolar plane Π, which intersects with image planes at lines l and $l^{\prime }$. $l^{\prime }$ is the epipolar line corresponding to $m_{i}$ on the image plane $I^{\prime }$, and $m_{i}^{\prime }$ is on the line $l^{\prime }$. Similarly, l is the epipolar line corresponding to $m_{i}^{\prime }$ on the image plane I. We define the constraint as epipolar geometry in binocular vision.

Suppose that the homogenous coordinates of the projection points can be expressed as:

(1) $$\begin{array}{l} m_{i}={\left[\begin{array}{ccc} u_{i} & v_{i} & 1 \end{array}\right]}^{T}\\ m_{i}^{\prime }={\left[\begin{array}{ccc} u_{i}^{\prime } & v_{i}^{\prime } & 1 \end{array}\right]}^{T} \end{array}$$

According to the epipolar geometry constraint:

(2) $${m_{i}^{\prime }}^{T}Fm_{i}=0.$$

The fundamental matrix ***F*** is defined as:

(3) $$F=\left[\begin{array}{ccc} F_{11} & F_{12} & F_{13}\\ F_{21} & F_{22} & F_{23}\\ F_{31} & F_{32} & 1 \end{array}\right].$$

By combining Equations (1)–(3), we develop the following equation:

(4) Uf=0

where:

(5) $$U=\left[\begin{array}{ccccccccc} u_{1}^{\prime }u_{1} & u_{1}^{\prime }v_{1} & u_{1}^{\prime } & v_{1}^{\prime }u_{1} & v_{1}^{\prime }v_{1} & v_{1}^{\prime } & u_{1} & v_{1} & 1\\ \vdots & \vdots & \vdots & \vdots & \vdots & \vdots & \vdots & \vdots & \vdots \\ u_{n}^{\prime }u_{n} & u_{n}^{\prime }v_{n} & u_{n}^{\prime } & v_{n}^{\prime }u_{n} & v_{n}^{\prime }v_{n} & v_{n}^{\prime } & u_{n} & v_{n} & 1 \end{array}\right].$$

(6) $$f={\left[\begin{array}{ccccccccc} F_{11} & F_{12} & F_{13} & F_{21} & F_{22} & F_{23} & F_{31} & F_{32} & 1 \end{array}\right]}^{T}.$$

The solution of the fundamental matrix is transformed into the process of solving the least squares of min‖Uf‖ with ‖f‖=1. Due to the influence of noise and mismatching, the fundamental matrix cannot be directly obtained by solving Equation (4). Therefore, in this case, we often use the RANSAC method to estimate the fundamental matrix.

The specific calculation process is as follows: Firstly, eight pairs of matching points are randomly selected from correspondences of feature point pairs, and Equation (4) is utilized to solve the fundamental matrix. Then, the points with geometric distances of less than the designed threshold value are judged as the inliers. By repeating the first two steps, the model with the largest number of inliers is selected as the fundamental matrix. The accuracy of the fundamental matrix estimation method based on RANSAC method depends on the proportion of inliers. When the ratio of inliers is low, it is difficult to find enough inlier sets to estimate the fundamental matrix, and the uneven distribution of matching points selected by random sampling will also affect the accuracy and stability of the estimation.

## 4. The Improved Fundamental Matrix Estimation Method of Planar Motion

### 4.1. Robust Null Space Estimation Method Based on Inlier Updating

The fundamental matrix of planar motion is analyzed in this section and the simplified fundamental matrix contains the constraint of coplanarity.

**Figure 2:**
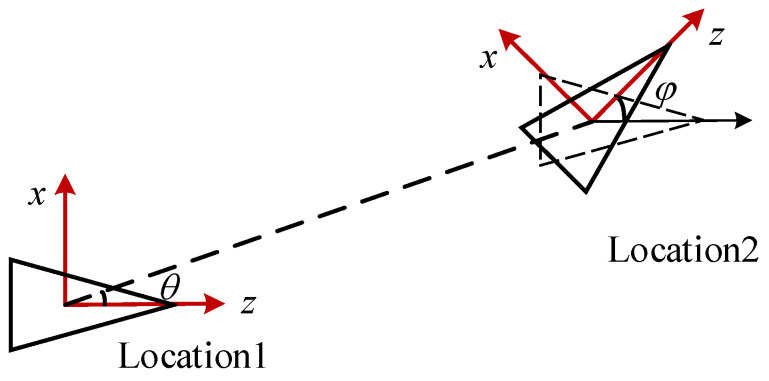
Camera motion scheme on the plane.

As shown in Figure 2, when the camera moves on the horizontal plane, the relative rotation and translation relationship between camera coordinate systems at location 1 and location 2 can be expressed as:

(7) $$R=\left[\begin{array}{ccc} \cos\varphi & 0 & \sin\varphi \\ 0 & 1 & 0\\ -\sin\varphi & 0 & \cos\varphi \end{array}\right],\;t=\left[\begin{array}{c} \sin\theta \\ 0\\ \cos\theta \end{array}\right].$$

where φ is the rotation angle and θ is the direction of translation.

The essential matrix ***E*** is defined as:

(8) E=t×R.

By substituting Equation (7) into Equation (8), the following equation can be obtained:

(9) $$E=\left[\begin{array}{ccc} 0 & -\cos\varphi & 0\\ \cos\left(\varphi -\theta \right) & 0 & \sin\left(\varphi -\theta \right)\\ 0 & \sin\theta & 0 \end{array}\right].$$

Assuming that the intrinsic matrix of the camera is ***K***, and the intrinsic parameters remain constant during camera movement, then ***K*** can be expressed as follows:

(10) $$K=\left[\begin{array}{ccc} f_{x} & s & x_{0}\\ 0 & f_{y} & y_{0}\\ 0 & 0 & 1 \end{array}\right].$$

Here, $f_{x}$ and $f_{y}$ are the normalized focal length on the X axis and Y axis; $\left(x_{0},y_{0}\right)$ is the principal point coordinate; *s* is the non-perpendicular factor between the X axis and Y axis.

According to the relationship between fundamental matrix and essential matrix, we obtain:

(11) $$F=K^{-T}EK^{-1}=\left[\begin{array}{ccc} 0 & f_{1} & f_{2}\\ f_{3} & 0 & f_{4}\\ f_{5} & f_{6} & f_{7} \end{array}\right].$$

For Equation (11):

(12) $$\begin{array}{l} d=1/f_{x}f_{y}\\ f_{1}=-d\cos\theta \\ f_{2}=dy_{0}\cos\theta \\ f_{3}=d\cos\left(\varphi -\theta \right)\\ f_{4}=df_{x}\sin\left(\varphi -\theta \right)-dx_{0}\cos\left(\varphi -\theta \right)\\ f_{5}=-dy_{0}\cos\left(\varphi -\theta \right)\\ f_{6}=df_{x}\sin\theta +dx_{0}\cos\theta \\ f_{7}=dx_{0}y_{0}\left(\cos\left(\varphi -\theta \right)-\cos\theta \right)-df_{x}y_{0}\left(\sin\left(\varphi -\theta \right)+\sin\theta \right) \end{array}$$

By substituting Equation (11) into Equation (2), we obtain:

(13) Mf=0.

where:

(14) $$M=\left[\begin{array}{ccccccc} u_{1}^{\prime }v_{1} & u_{1}^{\prime } & v_{1}^{\prime }u_{1} & v_{1}^{\prime } & u_{1} & v_{1} & 1\\ \vdots & \vdots & \vdots & \vdots & \vdots & \vdots & \vdots \\ u_{n}^{\prime }v_{n} & u_{n}^{\prime } & v_{n}^{\prime }u_{n} & v_{n}^{\prime } & u_{n} & v_{n} & 1 \end{array}\right],$$

(15) $$f={\left[f_{1},f_{2},f_{3},f_{4},f_{5},f_{6},f_{7}\right]}^{T}.$$

When there is no noise in the point correspondence, the rank of the null-space of ***M*** is 1. However, affected by noise and outliers, the rank of null-space does not equal one and ***M*** has no zero singular values. Motivated by the paper [40], to eliminate the outliers, let the matrix ***L*** represent the matrix ***M*** without noise, and the process of solving ***L*** can be converted into the following minimization:

(16) $$\begin{array}{l} \underset{W,L}{\arg\min}{\left\|W\left(M-L\right)\right\|}^{2}\\ \mathrm{rank}\left(L\right)=\mathrm{rank}\left(M\right)-1 \end{array}$$

In Equation (16), $W=diag\left(w_{1},w_{2},\cdots ,w_{n}\right)$ is an inlier updating matrix. If the *i*-th pair of matching points are inliers, set $w_{i}=1$, otherwise set $w_{i}=0$. Since ***L*** satisfies Lf=0, Equation (16) can be transformed into a minimization problem of Equation (17):

(17) $$\underset{W,f}{\arg\min}{\left\|WMf\right\|}^{2}.$$

Initially, let us assume that all the corresponding points are inliers, and ***W*** is a n×n identity matrix, ξ=Inf. Before iteration, ***W*** is updated according to the position similarity of feature points in the images. Subsequently, singular value decomposition is performed on $M^{T}WM$, and ***f*** is the eigenvector associated with the minimum singular value. If the condition $\varepsilon_{\max}>\xi$ is not met, ***W*** and ξ are updated according to Equation (18). Ultimately, ***W*** and ***f*** are substituted into the four-point iterative method as initial values:

(18) $$w_{i}=\{\begin{array}{cc} 1 & \varepsilon_{i}\leq \tau \\ 0 & \mathrm{otherwise} \end{array},\;\xi =\varepsilon_{\max},$$

(19) $$\varepsilon_{i}=\left(\frac{1}{\sqrt{{\left(Fm_{i}\right)}_{1}^{2}+{\left(Fm_{i}\right)}_{2}^{2}}}+\frac{1}{\sqrt{{\left(F^{T}m_{i}^{\prime }\right)}_{1}^{2}+{\left(F^{T}m_{i}^{\prime }\right)}_{2}^{2}}}\right)\left\|m_{i}^{\prime }Fm_{i}\right\|.$$

where $\varepsilon_{i}$ denotes the epipolar geometry distance; $\varepsilon_{\max}=Q_{25\%}\left(\varepsilon_{1},\cdots \varepsilon_{n}\right)$ is the lowest quartile of the epipolar geometry distance; and $\tau =\max(\varepsilon_{\max},\delta_{\max})$; $\delta_{\max}$ is the maximal geometric error.

### 4.2. Four-Point Iterative Method for Global Fundamental Matrix Estimation

In the estimation method based on traditional RANSAC method, the artificial threshold is usually utilized to iteratively update the model, and the fundamental matrix does not satisfy the rank constraint. Therefore, we used a singular value decomposition correction to solve the rank constraint problem.

In the four-point iterative method, primarily, the average value of the epipolar geometry distance calculated by the initial value ***f*** is taken as the threshold. Furthermore, four pairs of matching points are randomly selected from the inliers obtained by the initial value ***W*** and substituted into Equation (13):

(20) Af=0.

where:

(21) $$A=\left[\begin{array}{ccccccc} u_{1}^{\prime }v_{1} & u_{1}^{\prime } & v_{1}^{\prime }u_{1} & v_{1}^{\prime } & u_{1} & v_{1} & 1\\ \vdots & \vdots & \vdots & \vdots & \vdots & \vdots & \vdots \\ u_{4}^{\prime }v_{4} & u_{4}^{\prime } & v_{4}^{\prime }u_{4} & v_{4}^{\prime } & u_{4} & v_{4} & 1 \end{array}\right].$$

***A*** is a 4×7 matrix, $f_{1}^{\prime },f_{2}^{\prime },f_{3}^{\prime }$ are vectors that span the right null space of ***A***. Therefore, ***f*** can be expressed as:

(22) $$f=af_{1}^{\prime }+bf_{2}^{\prime }+cf_{3}^{\prime }.$$

These vectors are transformed into the fundamental matrixes and assume to be c=1:

(23) $$F=aF_{1}^{\prime }+bF_{2}^{\prime }+F_{3}^{\prime }.$$

When the camera moves on the plane, the intrinsic matrix of the camera ***K*** remains unchanged, and the rank of the fundamental matrix is 2. According to [26], the fundamental matrix satisfies:

(24) $$\begin{array}{l} \det\left(F\right)=0\\ \det\left(F+F^{T}\right)=0 \end{array}$$

By substituting Equation (23) into Equation (24), we obtain:

(25) $$C{\left[a^{3},a^{2}b,ab^{2},b^{3},a^{2},ab,b^{2},a,b,1\right]}^{T}=0.$$

where C is a parameter matrix constituted by the elements of $f_{1}^{\prime },f_{2}^{\prime },f_{3}^{\prime }$.

The parameters *a* and *b* can be solved by the minimum automatic generator method [41], and the fundamental matrix is estimated. Meanwhile, the number of matching points that satisfy the threshold is calculated and then these points are defined as a new inlier set. Eventually, through repeated use of the four-point iterative method, the model with the largest number of inliers is selected as the estimated fundamental matrix, which is stable and satisfies the rank constraint.

**Figure 3:**
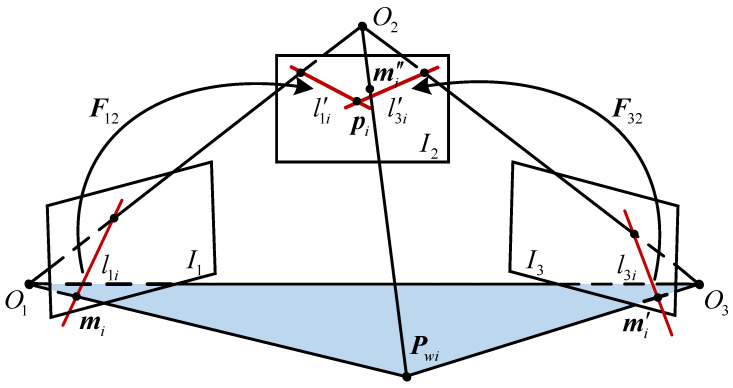
Epipolar geometry in triple-view.

The epipolar geometry in triple-view is shown in Figure 3, where the camera captures the object from different angles. The projection points of a 3D feature point $P_{wi}$ on image planes are $m_{i}$, $m_{i}^{\prime }$ and $m_{i}^{\prime \prime }$, respectively; $O_{1}$, $O_{2}$, $O_{3}$ are the optical centers of the cameras. Three images taken with public view as a group and $F_{12}$, $F_{32}$ are the fundamental matrices estimated by the four-point iterative method between the two images, respectively. Then, $m_{i},m_{i}^{\prime }$ in the first and third images are mapped to the epipolar lines $l_{1i}^{\prime }=F_{12}m_{i}$, $l_{3i}^{\prime }=F_{32}m_{i}^{\prime }$ in the intermediate image, and the intersection point of epipolar lines is set as $p_{i}$. Due to the influence of noise, there is a distance error between $p_{i}$ and $m_{i}^{\prime \prime }$. Therefore, $F_{12}$ and $F_{32}$ are simultaneously optimized by bundle adjustment:

(26) $$\underset{F_{13},F_{23}}{\arg\min}{\left\|p_{i}-m_{i}^{\prime \prime }\right\|}^{2}.$$

**Figure 4:**
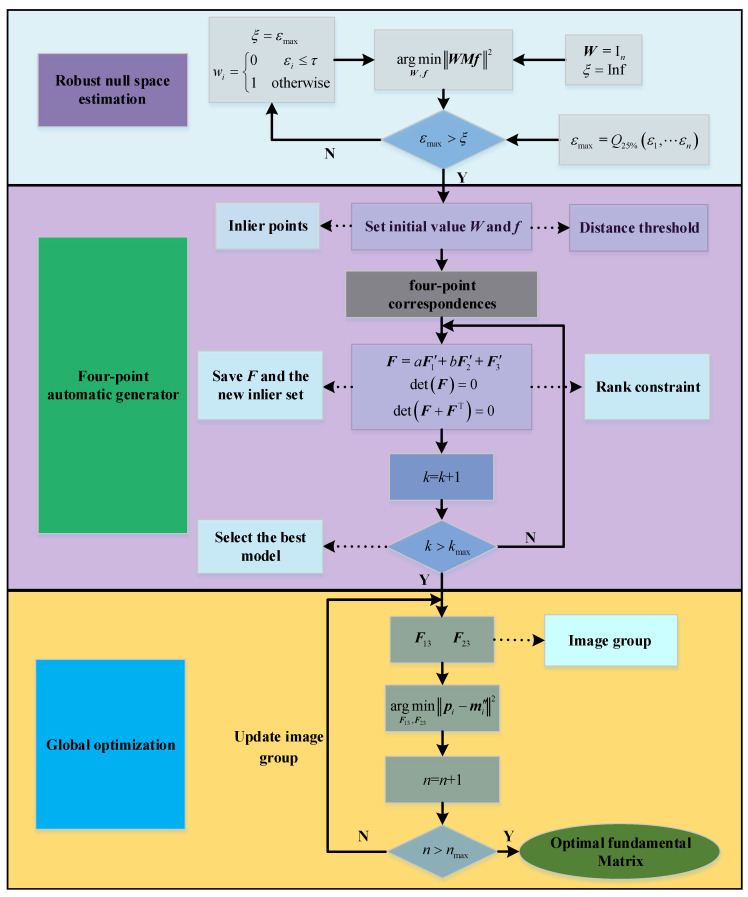
Flow chart of fundamental matrix estimation method.

Figure 4 shows the flow chart of the fundamental matrix estimation method in this paper. Firstly, the fundamental matrix is simplified by analyzing the motion characteristics of the camera, and the parameters to be solved are determined. Furthermore, the robust null space estimation method is applied to calculate the initial model of the fundamental matrix, and on this basis, a four-point automatic generator is derived. Ultimately, according to the epipolar geometry in triple-view, global optimization is carried out.

## 5. Experimental Results and Analysis

Two datasets were used in the experiment: one is the real dataset, and the other is a simulated dataset containing Gaussian noise and outliers. The proposed method is compared with ISSO [30], EGEC [31] and RANSAC [26] methods. In the subsequent experiments, the mean epipolar geometry distance and the mean distance between feature points and the intersection points of epipolar lines are used as the evaluation criteria to evaluate the accuracy of various methods.

### 5.1. Experiments on the Simulated Dataset

In the simulation test, we generated 300 pairs of corresponding points, which were uniformly distributed in the synthetic images. Gaussian noise with a mean value of 0, the standard deviation of σ and outliers with different proportions were added to simulate real-world conditions. We conducted 100 independent tests, taking the average value as the final result.

**Figure 5:**
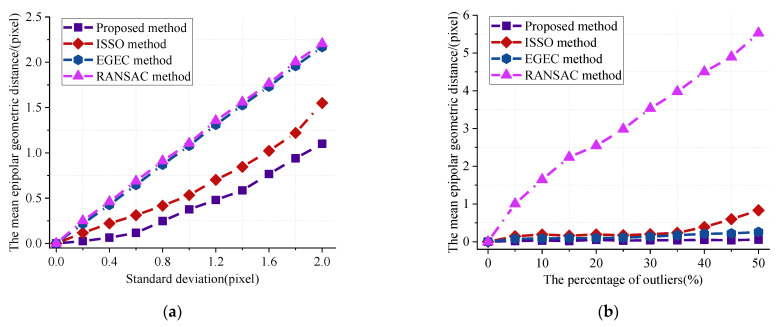
Comparisons of the epipolar geometric distances. (**a**) The mean geometric distance with varying Gaussian noise; (**b**) The mean geometric distance with varying outliers proportion.

In Figure 5a, the standard deviation of Gaussian noise increases from 0 to 2 without adding any outliers to the simulated data. The results show the epipolar geometric distance obtained by various fundamental matrix estimation methods under Gaussian noise. With the increase in noise standard deviation, the accuracy of the fundamental matrix estimated by all methods linearly decreases. Among them, the performance of the RANSAC method and EGEC method seems to be similar, and both of them sharply decline with the increase in noise. In contrast, the method proposed in this paper deteriorates slowly with the increase in noise. The experimental results in Figure 5b show that the other three methods have a stronger robustness to outliers than the RANSAC method when the corresponding points are noise-free. Moreover, it is clear that the epipolar geometry distance of the proposed method is essentially independent from the proportion of outliers.

**Figure 6:**
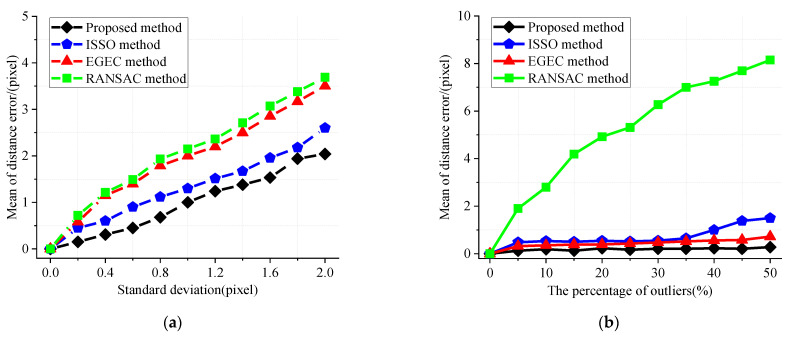
Comparisons of the distance between intersection points and feature points. (**a**) The mean distance with varying Gaussian noise; (**b**) The mean distance with varying outliers proportion.

In Figure 3, $p_{i}$ should coincide with $m_{i}^{\prime \prime }$. Gaussian noise and outliers are added to the simulation points in Figure 6a,b as Figure 5, respectively. As can be seen from Figure 6, under the same experimental conditions, the distance between $p_{i}$ and $m_{i}^{\prime \prime }$ obtained by the proposed method is the smallest, which indicates that this method has a good anti-interference performance and can better adapt to the uncertainty of noise and outliers.

In the second simulation experiment, the proportion of outliers is set at 10%, while Gaussian noise with mean value of 0 and standard deviation from 0 to 2 is added to the synthetic data. The experimental results of four methods for synthetic data are summarized in Table 1 and Table 2. When the ratio of outliers is fixed, the errors of the four estimation methods increase with the noise intensity. However, in comparison, the proposed method has the best performance when there are outliers and noises in the dataset.

### 5.2. Experiments on Real Dataset

**Figure 7:**
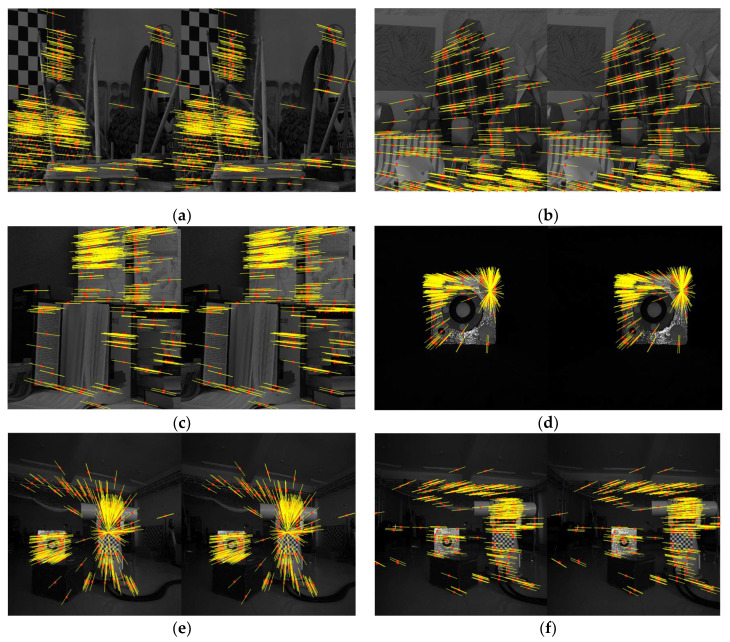
The epipolar geometry in different images. (**a**) Feature points and epipolar lines in Image1; (**b**) Feature points and epipolar lines in Image2; (**c**) Feature points and epipolar lines in Image3; (**d**) Feature points and epipolar lines in Image4; (**e**) Feature points and epipolar lines in Image5; (**f**) Feature points and epipolar lines in Image6.

Next, in order to verify the effectiveness of the proposed method under planar motion scenes, we randomly selected three images in the Middlebury dataset [42]. The images are taken from equally spaced viewpoints along the *x*-axis of camera coordinate system from left to right. Meanwhile, to further evaluate the performance of different fundamental matrix estimation methods in computer vision fields, we added a set of satellite images, as shown in Figure 7d, and a sequence of indoor images in the real experimental environment, as shown in Figure 7e,f.

The satellite was placed on the turntable and images were taken from nine perspectives from equiangular viewpoints. In the process, the camera was horizontal. The sequence of indoor images was collected by a camera mounted on a mobile robot horizontally, and the pose of the camera was provided by the OptiTrack system. Feature points were obtained by applying a Harris corner detector, and the correspondence of feature points was obtained by the optical flow method. The fundamental matrix was estimated by the proposed method, ISSO and EGEC methods.

Figure 7 shows the inlier feature points in the first and the intermediate images of the proposed method, which are marked as red ‘*‘. Additionally, the epipolar lines are recovered using the fundamental matrix estimated by the proposed method. As we can see, feature points fall exactly on the corresponding epipolar lines, indicating that the proposed method is relatively accurate for the estimation of the fundamental matrix. The mean epipolar geometry distance is shown in Table 3.

**Figure 8:**
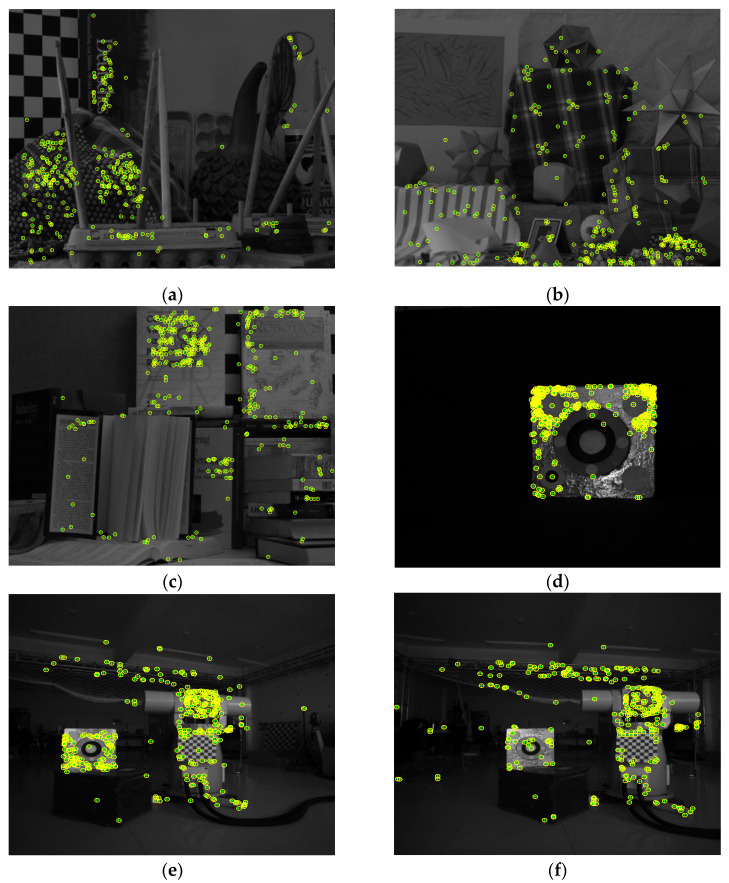
The feature points and intersection points in the intermediate images. (**a**) Feature points and intersection points in Image1; (**b**) Feature points and intersection points in Image2; (**c**) Feature points and intersection points in Image3; (**d**) Feature points and intersection points in Image4; (**e**) Feature points and intersection points in Image5; (**f**) Feature points and intersection points in Image6.

Figure 8 shows the inlier feature points in the intermediate images, which are labeled by ‘o’, and intersection points are labeled by ‘+’. It can be clearly seen that the intersection points and feature points calculated by the proposed method correctly coincide. Table 4 lists the mean distance between the intersection points and feature points calculated by each method. Compared with the other two methods, the proposed method is more robust.

From the above experiments, it can be included that the proposed fundamental matrix estimation method has a higher accuracy under different planar motion scenes. In order to display the application of the fundamental matrix estimation method in the field of 3D reconstruction and visual localization more intuitively, we designed the satellite reconstruction and indoor localization experiments.

**Figure 9:**
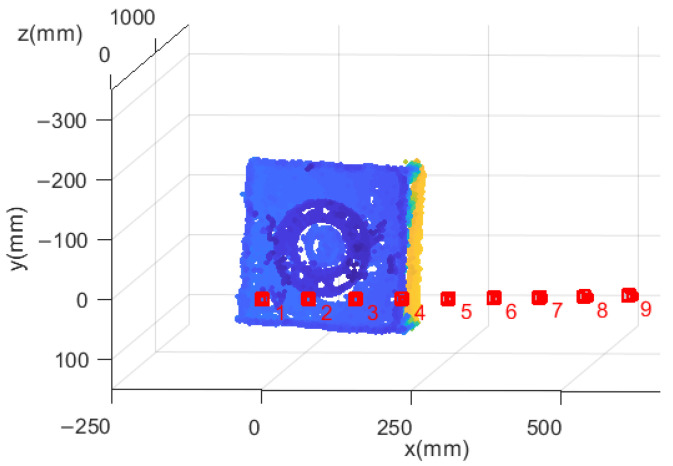
Satellite 3D feature point model.

In 3D reconstruction, the static measurement and dynamic measurement are devised to verify the reconstruction accuracy of feature points by calculating the rotation angle of the satellite. Firstly, we extract the feature points in the satellite dataset images and obtain the correspondences of feature points. The camera coordinate system in the first image is defined as the world coordinate system by default. The fundamental matrix estimation method proposed in this paper and the ISSO method, respectively, are used to reconstruct the feature points on the satellite and establish the 3D feature point models of the satellite. Figure 9 shows the satellite 3D feature point model obtained by our method and the relative position relationship between camera coordinate systems.

Afterwards, the turntable is rotated every 2° for a total of 10°, and the control accuracy is 0.010°. The satellite 3D feature point models obtained by the proposed method and ISSO method are used to measure the static rotation angle by applying the perspective-n-point algorithm. The measured static errors are shown in Table 5. It can be seen that the error gradually increases with the increase in the rotation angle, and the estimation method proposed in this paper can control the maximum error of the rotation angle within 0.104°, which is better than the result of the ISSO method (0.138°).

**Figure 10:**
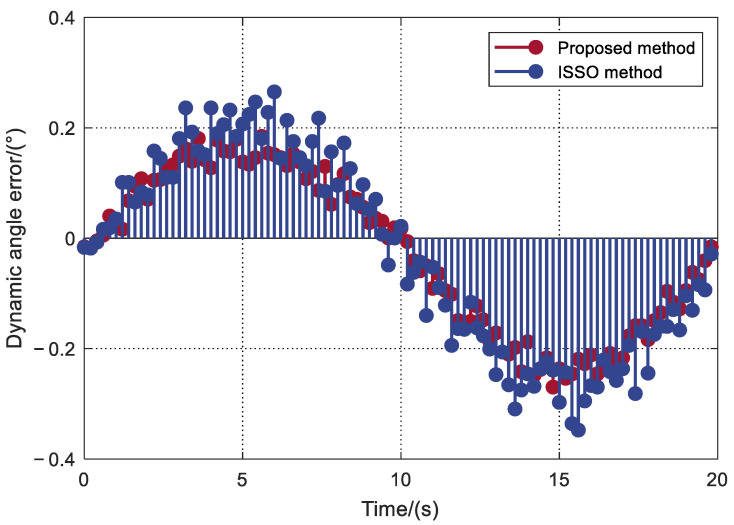
Dynamic rotation angle error.

The motion trajectory of the turntable is a cosine curve with amplitudes rising from 0° to 10°, and the average speed of the turntable is 1°/s. Figure 10 shows the dynamic errors of the rotation angle in one cycle of motion. We can see that the proposed method in this paper can control the dynamic error of satellite up to 0.273°, but the dynamic error of ISSO method is relatively high.

**Figure 11:**
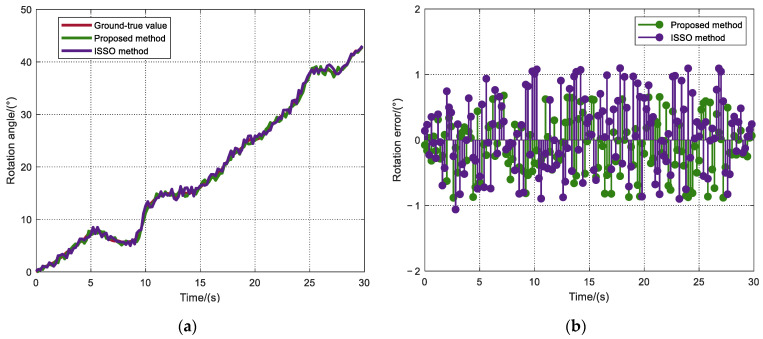
Ground-true value and error of rotation angle. (**a**) The ground-true value of the rotation angle; (**b**) Error of rotation angle.

In visual localization, after we obtain the point correspondence, the proposed method and ISSO method are used to estimate the fundamental matrix between the indoor images. The rotation angle of the camera calculated by the fundamental matrix at different moments is used to verify the accuracy of visual localization. We calculate the camera rotation angle in 30 s with the angle amplitudes rising from 0° to 43°. Figure 11a,b show the ground-true value and error of the rotation angle, respectively. In Figure 11b, the average rotation error obtained by the ISSO method is 0.517°; however, the error obtained by the proposed method is 0.362°. It can be seen that the error distribution of the proposed method is more concentrated than that of the ISSO method.

## 6. Conclusions

In this paper, we propose a robust method for estimating the fundamental matrix from point correspondence in multi-view images. Firstly, stable and reliable initial values are obtained by combining the outlier elimination with the fundamental matrix estimation. Even if there is a large number of outliers, the calculated values will soon become stable. Then, the rank constraint is introduced, and we solve the problem of reducing the accuracy of the fundamental matrix in a traditional singular value decomposition correction. The global optimization function under triple-view is constructed, which further improves the estimation accuracy of the fundamental matrix. The experimental results show that the proposed method is more accurate and robust than traditional methods. It can solve the fundamental matrix estimation problem under planar motion scenes. This kind of method is beneficial for the research of high precision non-cooperative target pose measurement and has important significance for the planning research of mobile robots.

## Figures and Tables

**Table 1:** The mean epipolar geometric distance of synthetic data (pixel).

Noise Level	Proposed Method	ISSO Method	EGEC Method	RANSAC Method
0	0.031	0.191	0.086	1.645
0.2	0.056	0.308	0.303	1.896
0.4	0.094	0.412	0.511	2.105
0.6	0.147	0.503	0.731	2.333
0.8	0.277	0.607	0.955	2.556
1	0.406	0.724	1.164	2.75
1.2	0.51	0.893	1.396	3.001
1.4	0.616	1.036	1.612	3.206
1.6	0.797	1.213	1.817	3.411
1.8	0.971	1.411	2.045	3.65
2	1.132	1.741	2.254	3.851

**Table 2:** The mean distance between feature point and intersection point of synthetic data (pixel).

Noise Level	Proposed Method	ISSO Method	EGEC Method	RANSAC Method
0	0.191	0.534	0.354	2.812
0.2	0.345	0.983	0.934	3.524
0.4	0.498	1.135	1.502	4.013
0.6	0.641	1.432	1.752	4.292
0.8	0.875	1.652	2.141	4.733
1	1.19	1.836	2.352	4.95
1.2	1.43	2.047	2.553	5.163
1.4	1.574	2.207	2.851	5.514
1.6	1.723	2.491	3.207	5.87
1.8	2.13	2.713	3.524	6.183
2	2.231	3.135	3.857	6.488

**Table 3:** The mean epipolar geometric distance of real images (pixel).

Data	Proposed Method	ISSO Method	EGEC Method
Image1	0.618	0.992	1.615
Image2	0.815	1.241	1.826
Image3	1.038	1.41	2.346
Image4	0.509	0.976	1.587
Image5	1.161	1.817	2.455
Image6	1.291	2.019	2.532

**Table 4:** The mean distance between feature point and intersection point of real images (pixel).

Data	Proposed Method	ISSO Method	EGEC Method
Image1	1.513	2.162	2.893
Image2	1.775	2.685	3.381
Image3	2.309	2.787	3.502
Image4	1.402	2.054	2.586
Image5	2.271	3.017	3.883
Image6	2.406	3.137	4.038

**Table 5:** Static rotation angle error (°).

Data	Measured Angle		Standard Angel	Absolute Error	
	Proposed Method	ISSO Method		Proposed Method	ISSO Method
1	0.013	0.023	0	0.013	0.023
2	2.034	1.942	2	0.034	0.058
3	3.949	3.923	4	0.051	0.077
4	6.075	6.086	6	0.075	0.086
5	8.091	7.887	8	0.091	0.113
6	9.896	10.138	10	0.104	0.138

## Data Availability

Not applicable.
